# 热消融治疗原发性和转移性肺部肿瘤的专家共识（2014年版）

**DOI:** 10.3779/j.issn.1009-3419.2014.04.01

**Published:** 2014-04-20

**Authors:** 欣 叶, 中国抗癌协会肿瘤微创治疗专业委员会肺癌微创综合治疗分会

**Affiliations:** 1 510060 广州，中山大学肿瘤防治中心影像介入中心 Department of Oncology, Shandong Provincial Hospital Affliated to Shandong University, Ji'nan 250014, China; 2 250014 济南，山东大学附属省 立医院肿瘤科 Imaging Intervention Center, Cancer Center, Sun Yat-sen University, Guangzhou 510060, China

## 1 前言

在世界范围内肺癌均居癌症发病率和死因之首，全球每年发病约2, 500, 000人，每年有超过1, 600, 000人死于肺癌^[1]^。在我国肺癌的发病形势更加严峻，据《2012年中国肿瘤年报》^[2]^报导我国肺癌年发病率：57.63/10万，肺癌年死亡率为48.87/10万，绝对数均排在世界第一。对于早期非小细胞肺癌（non-small cell lung cancer, NSCLC）在没有转移的情况下外科切除是治愈的主要手段^[3]^，但是由于各种原因，大约80%的肺癌无法通过手术切除治疗。对于无法手术切除的多数肺癌患者在传统的放化疗中获益有限，因此许多新的局部治疗方法应运而生，包括局部消融治疗等。局部热消融术作为一种微创技术已经应用在早期肺癌的治疗，每年治疗肺癌患者的例数迅速增加^[4]^。肺部转移瘤在临床上十分常见，目前已证实经皮热消融也可以有效地治疗肺部转移瘤^[5]^。为了融合国内外先进诊疗技术理念并与国际接轨，推动我国临床肿瘤微创技术的发展，提高肺部恶性肿瘤多学科综合治疗的水平，加强国内各医院肺癌微创诊疗专业之间的交流，更好地规范热消融治疗肺部恶性肿瘤的操作技术，“中国抗癌协会肿瘤微创治疗专业委员会肺癌微创综合治疗分会”组织多学科专家参与制定了“热消融治疗原发性和转移性肺部肿瘤的专家共识”，以便为临床实践提供参考。

2013年8月25日在郑州召开的“第九届中国肿瘤微创治疗学术大会”上，在吴沛宏教授的提议下，由范卫君、叶欣教授发起，经过与国内多名专家反复研究和磋商后，成立了“中国抗癌协会肿瘤微创治疗专业委员会肺癌微创综合治疗分会”（简称“分会”）筹备组。2013年10月25日-26日在济南“分会”正式成立，并召开了“第一届中国肺癌微创综合治疗学术交流会”。“分会”由来自全国22个省市自治区112名专家组成，分跨专业有肿瘤科、放射介入科、胸外科、放疗科、呼吸科、生物治疗和基础研究等。在此次会议的年度工作会议上，一致同意由范卫君和叶欣教授牵头起草“热消融治疗原发性和转移性肺部肿瘤的专家共识”（简称“共识”）。“共识”草案形成后，在2013年12月-2014年2月期间反复多次通过电子邮件的形式广泛争取了“分会”各位委员的意见，根据各位委员提出的宝贵意见，先后3次修改“共识”草案。2014年3月1日在济南召开了“共识”定稿会，国内有关肿瘤微创诊治领域的18位知名专家出席会议。按照“尊重循证医学证据，融合国际诊疗理念，体现我国特色，便于临床实践和操作”的原则，在会上各位专家言语锋锐、各抒己见、集思广益、求同存异、删繁就简，最终达成了本“共识”。

## 2 肿瘤热消融的概念

肿瘤热消融是针对某一脏器中特定的一个或多个肿瘤病灶，利用热产生的生物学效应直接导致病灶组织中的肿瘤细胞发生不可逆损伤或凝固性坏死的一种治疗技术。在我国属于第三类医疗技术（卫办医政发[2009]84号）。

## 3 局部热消融技术

热消融治疗技术，主要包括射频消融术（radiofrequency ablation, RFA）、微波消融术（microwave ablation, MWA）、冷冻消融术（cryoablation）、激光消融术（laser ablation）和高强度聚焦超声（high-intensity focused ultrasound, HIFU）^[6]^，激光消融术和HIFU在我国较少用于肺部肿瘤的消融治疗。

### 3.1 射频消融

RFA是目前治疗实体瘤应用最广泛的消融技术，其原理是将射频电极穿刺入肿瘤组织中，在200 kHz-650 kHz的高频交变电流作用下，肿瘤组织内的离子相互摩擦、碰撞而产生热生物学效应，局部温度可达60 ℃-120 ℃，当组织被加热至60 ℃以上时，可引起细胞凝固性坏死，RFA消融体积取决于局部射频消融产生的热量传导与循环血液及细胞外液间的热对流^[6-9]^。2007年12月美国FDA批准了RFA可以用于肺部肿瘤的治疗^[9]^，2009年以来非小细胞肺癌NCCN指南、中国《原发性肺癌诊疗规范（2011年版）》（卫办医政发[2011]22号）均推荐RFA可以用于早期不能耐受手术切除肺癌患者的治疗。

### 3.2 微波消融

MWA一般采用915 MHz或2, 450 MHz两种频率。在微波电磁场的作用下，肿瘤组织内的水分子、蛋白质分子等极性分子产生极高速振动，造成分子之间的相互碰撞、相互摩擦，在短时间内产生高达60 ℃-150 ℃的高温，从而导致细胞凝固性坏死^[10-12]^。由于辐射器将微波能集中在一定范围内，故而能有效地辐射到所需靶区，微波热辐射在肺内有更高的对流性和更低的热沉降效应^[13-17]^。

### 3.3 冷冻消融

氩-氦冷冻消融是目前较成熟的冷冻治疗技术。其原理是高压氩气可以冷却至零下140 ℃，氦气可使零下140 ℃迅速上升至零上20 ℃-40 ℃，通过这种温度梯度的变化可以导致^[18-20]^：①靶组织蛋白质变性；②细胞内外渗透压改变和“结冰”效应造成细胞裂解；③微血管栓塞引起组织缺血坏死等。用计算机断层扫描（computed tomography, CT）或磁共振成像（magnetic resonance imaging, MRI）观察到的“冰球”可以直接将消融区域与肿瘤边界进行区分，使手术者能够对临近重要结构的肿瘤进行相对安全的治疗，可以测定冷冻损伤的边界，这一边界大致在冰球最外缘内侧4 mm-6 mm范围内^[19, 20]^。

虽然三种消融技术均能应用于肺部肿瘤的局部治疗，但三者各有优势，在临床实践中要合理选择消融方式，取长补短，以达到满意的治疗效果。对于直径≤3 cm的肿瘤，三种消融方式均获得良好的治疗效果。而对于直径 > 3 cm，尤其是 > 5 cm的肿瘤，MWA因其消融时间短、消融范围广，明显优于其他两种消融方式，且MWA受到血流灌注的影响小，更加适合治疗邻近大血管的肿瘤。而冷冻消融形成的“冰球”边界清晰，易于监测，可应用于邻近危险脏器的肺部肿瘤。冷冻消融较少引起局部疼痛，对于肿瘤距离胸膜≤1 cm或有骨转移引起骨质破坏的肿瘤患者，冷冻消融明显优于MWA和RFA。但冷冻消融在治疗过程中消耗患者血小板，对于凝血功能差的患者，应避免使用冷冻消融。RFA电极的适形性好，可以通过调节消融电极来保护邻近脏器。

## 4 操作平台

### 4.1 影像引导

经皮热消融治疗的影像引导技术有：CT、MR、超声等。CT是肺部肿瘤消融治疗最常用的影像引导技术，其次是MR。对于用超声能观察到肿瘤全貌的靠近胸壁或与胸壁粘连的肿瘤，可以用超声引导。

### 4.2 开胸或电视胸腔镜辅助下

一般用于：①肺部肿瘤邻近致命的结构如大血管、肺门或心脏；②在开胸后发现肺部肿瘤不能够切除的情况下^[21]^。

## 5 肺部肿瘤热消融的适应证和禁忌证

### 5.1 完全性消融（complete ablation）的适应证

完全性消融是指通过热消融治疗，使局部肿瘤病灶组织完全坏死，并有可能达到治愈的效果。

#### 5.1.1 原发性周围型肺癌^[22-27]^

① 患者因心肺功能差或高龄不能耐受手术切除；②拒绝行手术切除；③其它局部治疗复发后的单发病灶（如适型放疗后）。肿瘤最大径≤3 cm，且无其他部位的转移。

#### 5.1.2 肺部转移瘤^[24, 25, 28-33]^

某些生物学特征显示预后较好的肺内转移瘤（如肉瘤、肾癌、结直肠癌、乳腺癌、黑色素瘤和肝细胞癌）。如果原发病能够得到有效治疗，可进行肺转移瘤的消融治疗。单侧肺病灶数目≤3个（双侧肺≤5个），多发转移瘤最大肿瘤的最大直径≤3 cm，单侧单发转移瘤的最大直径≤5 cm，且无其他部位的转移。对于双侧肺肿瘤，不建议双侧同时进行消融治疗。

### 5.2 姑息性消融（palliative ablation）的适应证

治疗的目的在于最大限度减轻肿瘤负荷、缓解肿瘤引起的症状和改善患者生活质量，对于达不到完全性消融条件的患者，其适应证可以较完全性消融适当放宽。如肿瘤最大径 > 5 cm，可以进行多针、多点或多次治疗，或与其他治疗方法联合应用。如肿瘤侵犯肋骨或胸椎椎体引起的难治性疼痛，对肿瘤局部骨侵犯处进行消融，即可达到止痛效果^[25, 34-37]^。

### 5.3 热消融禁忌证

因为肺癌患者对经皮热消融治疗具有良好的耐受性，除无法纠正的凝血障碍性疾病以外，肺部肿瘤局部热消融的绝对禁忌证较少^[7, 24, 38-42]^。

#### 5.3.1

病灶周围感染性及放射性炎症没有很好控制者，穿刺部位皮肤感染、破溃。

#### 5.3.2

有严重出血倾向、血小板 < 50×10^9^/L和凝血功能严重紊乱者（凝血酶原时间 > 18 s，凝血酶原活动度 < 40%）。抗凝治疗和/或抗血小板药物应在经皮消融前至少停用5 d-7 d。

#### 5.3.3

消融病灶同侧恶性胸腔积液没有很好控制者。

#### 5.3.4

肝、肾、心、肺、脑功能严重不全者，严重贫血、脱水及营养代谢严重紊乱，无法在短期内纠正或改善者，严重全身感染、高热（> 38.5 ℃）者。

#### 5.3.5

晚期肿瘤患者Karnofsky功能状态（Karnofsky performance status, KPS）评分 < 70分或美国东岸癌症临床研究合作组（Eastern Cooperative Oncology Group Performance Status, ECOG）评分 > 3分。

## 6 术前准备

### 6.1 患者的评估及影像学检查

要通过认真复习病史、体格检查及近期的影像资料来评估患者的热消融适应证。适应证的选择建议多学科共同讨论做出决定，并有消融手术前讨论记录。胸部强化CT（2周内）为消融治疗前评估的关键影像学检查，通过CT观察肿瘤的大小、位置及其与临近重要脏器、血管、气管或支气管的关系。完善相关分期检查，有条件者建议行正电子发射断层显像/计算机断层成像（positron emission tomography/computed tomography, PET-CT）检查排除或发现远处转移^[43]^。

### 6.2 各项实验室检查

实验室检查应包括：血常规、大小便常规、凝血功能、肝肾功能、血糖、肿瘤标记物、血型等检查，心电图、心脏彩超（高龄患者可选）、肺功能等。

### 6.3 病理检查

对原发性肺癌，消融治疗前行经皮病灶穿刺活检或者纤维支气管镜检查以明确诊断。当转移病灶不典型时建议消融治疗前对病灶进行活检^[24, 41-44]^。

### 6.4 药品及监护设备准备

术前应准备麻醉、镇痛、镇咳、止血、扩血管、降压等药物、抢救药品及设备。

### 6.5 患者准备

#### 6.5.1

患者及家属（被委托人）签署知情同意书。

#### 6.5.2

局部麻醉前4 h禁食，全身麻醉前12 h禁食、前4 h禁水。

#### 6.5.3

手术区必要时备皮。

#### 6.5.4

建立静脉通道。

#### 6.5.5

术前口服镇咳剂。

## 7 麻醉、消融中及消融后的监护

### 7.1 麻醉与消毒

根据患者的状况，可以采用全身麻醉或局部麻醉进行消融手术^[45, 46]^，要执行无菌操作技术规范。

### 7.2 消融中及消融后的监护

选择合适的消融技术后，CT是最常用和最准确的影像引导方式之一，操作过程是将热消融电极（或天线或探针），在CT引导下通过皮肤直接穿刺入靶组织中。通过多平面的CT影像确认消融电极（或天线或探针）处于正确位置后，进行消融治疗（肺组织消融参数的选择可以根据不同设备生产商推荐的参数进行适当调整）。根据肿瘤的大小和部位可采用多种模式进行消融治疗：①单次单点完成消融治疗（如直径≤3 cm者）；②单次多点完成消融治疗（如直径3 cm-5 cm者）；③多电极（多天线或多探针）单次多点或多次多点完成消融治疗（如直径 > 5 cm者或姑息消融）。热消融过程中，由于热消融对肿瘤周围肺组织的损伤，在肿瘤周围可出现不透明高密度区—称为毛玻璃样影（ground-glass opacity, GGO），当靶组织GGO的周边界限大于消融前肿瘤的5 mm以上时，消融电极（或天线或探针）可以拔出，拔出消融电极（或天线或探针）时要注意消融穿刺针道。消融过程结束时要再次CT扫描（范围要大，最好是全肺扫描），以观察是否有并发症的发生，同时初步评价操作技术的成功情况。

消融过程需要监测心率、血压和血氧饱和度，同时要观察患者的呼吸、疼痛、咳嗽、咯血等情况，必要时应对症处理。术后建议监测生命体征，24 h-48 h后拍胸片或CT扫描，观察是否有并发症的发生（如无症状性气胸或胸腔积液）。

## 8 随访及疗效评估

### 8.1 随访

术后前3个月，每个月复查一次胸部增强CT。以后每3个月复查胸部增强CT或PET-CT和肿瘤标志物。主要观察局部病灶是否完全消融、肺内有无新发病灶及肺外转移等。胸部增强CT是目前评价消融效果的标准方法，有条件的可使用PET-CT，PET-CT/强化CT两者相结合可以更准确地判断消融后的疗效^[24, 43, 47]^。

### 8.2 术后影像学表现及疗效评估

#### 8.2.1 CT疗效评估

##### 8.2.1.1 影像学表现

消融后强化CT扫描显示的变化规律为：消融后1个月-3个月内病灶增大，3个月后病灶保持稳定或逐渐缩小^[47, 48]^。①早期改变（1周内）：病灶内可出现蜂窝状或空洞样低密度影，消融肿瘤周边为不同衰减程度的同心圆包围，称为“帽徽”（cap badge）征象（此征象在消融后24 h-48 h更加明显）。CT值减低，病灶较消融前增大，周边呈现GGO样反应带，一般认为GGO应超出肿瘤周边边缘至少5 mm可达到肿瘤完全消融^[24, 49-51]^；②中期改变（1周-3月内）：消融区可持续增大，其周边可能出现环绕清晰锐利的强化环，称为“蛋壳”（egg shell）征象。③后期改变（3月后）：与基线（一般以病灶消融后1个月时的CT表现为基线^[29]^）比消融区在3个月后病灶保持稳定，以后的CT随访过程中病灶区域有几种不同的演变模式：如纤维化、空洞、结节、肺不张、消失等^[50]^。

##### 8.2.1.2 局部疗效评估

以消融后1个月时的病灶为基线判断疗效。①完全消融（出现下列表现任何一项）：病灶消失；完全形成空洞；病灶纤维化，可为疤痕；实性结节缩小或无变化，但CT扫描无造影剂强化征象；肺不张，肺不张内的病灶CT扫描无造影剂强化征象；②不完全消融（出现下列表现任何一项）：空洞形成不全，有部分实性或液性成分，且CT扫描有造影剂强化；部分纤维化，病灶部分纤维化仍存有部分实性成分，且实性部分CT扫描有造影剂强化；实性结节，大小无变化或增大，且伴CT扫描造影剂有强化征象。

#### 8.2.2 PET-CT

PET-CT可能是消融后判断局部疗效最准确的手段，但消融后3个月内行PET-CT检查假阳性率较高。PET-CT由于种种原因尚未在肿瘤消融领域中广泛应用，建议有条件的机构使用PET-CT随访肺肿瘤消融后的疗效^[52-55]^。

### 8.3 临床疗效评估

在判断局部疗效的基础上，定期随访，消融后3个月，以后每隔3个月复查一次强化CT，1年后每6个月复查一次强化CT，共复查2年-3年。观察患者生存质量的改善情况（如疼痛缓解情况，可以用疼痛评分评估）。随访患者生存时间，生存时间是最重要的临床疗效指标，要记录患者1年、2年、3年、5年的生存情况。

## 9 并发症及处理

经皮肺肿瘤消融术是一种相对安全的局部治疗手段，其并发症的发生情况，根据介入放射学学会影像引导肿瘤消融国际工作组分类进行报告^[56]^。①严重并发症：导致死亡或者致残，需要住院或者临床处理，提升护理级别或延长住院时间，包括输血、胸腔闭式引流。死亡需要说明与消融之间的关系；②轻微并发症：其他并发症，包括不需要引流的气胸等；③副反应：指伴随治疗出现的不良结果，一般经常发生，但很少造成实质性的损害，包括疼痛、胸膜反应、少量肺内出血、血痰、少量胸腔积液、消融后综合征等。按照发生时间分为即刻并发症（消融后 < 24 h）、围手术期并发症（消融后24 h-30 d）及迟发并发症（消融后 > 30 d）。

### 9.1 疼痛

在局麻条件下手术，一般均有不同程度的疼痛（尤其是临近胸膜的病变行消融治疗时常常需要止痛治疗）。如果疼痛剧烈，可以加大阿片类止痛药物的用量，同时可以给予适量镇静剂。手术后疼痛一般为轻度疼痛，可持续数天，也有人持续1周-2周，很少出现中度以上的疼痛，可以用非甾体类药物止痛。

### 9.2 消融后综合征

约2/3患者可能发生，是由于坏死物质的吸收和炎性因子的释放引起。主要症状为低热、乏力、全身不适、恶心、呕吐等，一般持续3 d-5 d，少部分可能会持续2周左右。这种情况对症处理即可，必要时除给予非甾体类药物外，可以适量短时应用小剂量糖皮质激素，同时要加强支持治疗。

### 9.3 气胸

气胸是消融后最常见的并发症，发生率为10%-60%^[57, 58]^。气胸更常见于以下情况：肺气肿、男性、年龄 > 60岁、肿瘤 < 1.5 cm、肿瘤位于肺下叶、单发肿瘤穿刺肺组织次数 > 3次、消融多个肿瘤穿刺次数多、消融路径穿过肺组织的长度较长或者穿过较大的叶间裂^[59-61]^。大部分气胸容易治疗，或者是自限性的，不需要治疗即可自愈，需要胸腔闭式引流的不超过10%。如果患者经过胸腔闭式引流仍然有气体漏出，可以持续负压吸引、行胸膜固定术、气管镜下注入硬化剂、气管内置入阀门等^[62]^。另外，要注意迟发性气胸的发生，一般认为消融后72 h后发生的气胸称为迟发性气胸^[63]^。

### 9.4 胸腔积液

消融后经常可以见到少量胸腔积液，发生率为1%-60%^[57]^，被认为是机体对热损伤的交感反应，需要穿刺/置管引流的胸腔积液占1%-7%。导致胸腔积液发生的危险因素有：大病灶、一次消融多个病灶、病灶靠近胸膜（< 10 mm）、消融时间长等^[59]^。

### 9.5 出血

消融中出血的发生率在3%-8%^[56, 57]^，但是大咯血的发生率极低^[57, 64, 65]^。如果出现中等以上的咯血后应立即消融，同时静脉输注止血药。由于消融本身可以使血液凝固，随着消融治疗的进行出血会逐渐停止，故在具体消融治疗过程中大出血的发生率并不高。在穿刺过程中应尽量避免穿刺到较大血管或者不张的肺组织等。术后咯血，多具有自限性，可持续3 d-5 d。保守治疗无效者，可行介入栓塞治疗或剖胸探查。

### 9.6 胸膜反应

消融过程中刺激了支配壁层胸膜的迷走神经，兴奋的迷走神经可使心率减慢、甚至心跳停止。出现这种情况要暂停消融，要充分局部麻醉，并适当应用阿托品、镇静剂等药物。

### 9.7 感染

消融手术引起的肺部感染的发生率为1%-6%^[57, 64-66]^。术前30 min-1 h可以预防性应用抗生素，24 h内再用一次。在下列情况下消融手术后预防性应用抗生素可以适当延长到48 h-72 h：老年人 > 70岁、长期慢性阻塞性肺气肿、糖尿病控制欠佳、肿瘤 > 4 cm、免疫力低下等。若消融手术后5 d体温仍然 > 38.5 ℃，首先要考虑肺部感染，要根据痰液、血液或脓液培养的结果调整抗生素。如果发生肺部或胸腔脓肿可以置管引流并冲洗。另外，放疗后患者易发生间质性肺炎，在此基础上行消融术者更易继发感染，要引起注意^[66]^。

### 9.8 其他少见并发症

支气管胸膜瘘、急性呼吸窘迫综合症、非靶区热灼伤或冻伤、冷休克、血小板降低、肿瘤针道种植、神经损伤（臂丛、肋间、膈、喉返等神经）、肺栓塞、空气栓塞、心包填塞等均有个案报道，需个别特殊处理。

## 10 消融和其他治疗联合

消融与其他方法进行联合治疗是目前许多肿瘤研究的重要内容之一，包括消融与外科、化疗、放疗和分子靶向药物等的联合。消融与放疗可以提高肿瘤的局部控制率，延长患者的生存期，而副反应无明显增加^[67-69]^。对于NSCLC消融与化疗结合的研究相对较少，但是对于提高肿瘤的局部控制率、延长患者的生存期方面有一定益处^[70-73]^。

## 11 结语

关于肺部肿瘤的治疗，微创治疗是未来发展的方向之一，尤其是影像引导下的经皮热消融技术在治疗肺部肿瘤方面具有：创伤小、疗效明确、安全性高、患者恢复快、操作相对简单、适应人群广等特点。最近研究^[74, 75]^表明：经皮热消融治疗不能耐受手术切除早期NSCLC患者（肿瘤直径2 cm-3 cm）的1年、3年和5年的生存率分别达到97.7%、72.9%和55.7%，且死亡率小于1%。这些临床证据让我们相信未来这一技术会在肺部肿瘤的综合治疗中得到越来越广泛的应用，其地位有可能成为继手术、放疗、化疗之后的一种新的治疗模式。但是从临床实践的角度看，有关热消融技术治疗原发性和转移性肺部恶性肿瘤患者的例数与手术、放疗和化疗相比相对较少^[76-80]^，需要进一步开展工作以改变传统肿瘤工作者对热消融技术的认知，使得该治疗方法得以普及和规范化应用。

目前热消融技术治疗原发性和转移性肺部恶性肿瘤还存在许多问题：①热消融技术已经成为肺部肿瘤多学科综合治疗领域的重要手段，特别是对于早期不能耐受外科手术切除的周围型肺癌患者有可能成为首选，但是尚缺乏大规模的、多中心的、随机的、前瞻性的临床研究；②缺乏与其他传统治疗手段（如立体定向放射治疗）的前瞻性的、多中心的临床比较研究；③热消融与其他治疗手段（如放疗、化疗和分子靶向药物治疗等）联合应用的临床研究相对较少^[81-83]^；④如何提高局部完全消融率，降低局部复发，也是今后工作的方向之一；⑤作为我国的“三类医疗技术”，由于治疗设备的生产厂家不同，设备性能之间的差异，再加上该专业刚刚兴起，治疗人员的专业化水平参差不齐，现在很难形成公认的治疗规范；⑥应用热消融技术治疗后的疗效判断有时较难与现行的国际标准接轨，使用现有的影像学手段有时较难真实反映出热消融技术治疗后的疗效，因此，制定公认的、符合热消融技术自身规律的疗效判断标准还需要进行艰苦的工作；⑦姑息消融在肺癌综合治疗的位置还有待于进一步探讨；⑧基础研究相对滞后，如复杂热场分布、对机体免疫的影响等等。

本“共识”虽然借鉴了许多国际指南和国内外的最新研究进展，也经过了多次认真讨论和反复修改，仍难免存在不足和局限性，因此，需要在以后的临床实践中不断补充，动态完善，以期制定出与国际接轨并且符合我国国情的热消融技术临床指南，以推动热消融技术应用、研究和水平提高，为我国人民和全人类的卫生健康事业作出积极贡献。
